# “Smart agriculture: a climate-driven approach to modelling and forecasting fall armyworm populations in maize using machine learning algorithms”

**DOI:** 10.3389/fpls.2025.1636412

**Published:** 2025-10-30

**Authors:** Vani Sree Kalisetti, Upendhar Sudharshanam, Nagesh Kumar Mallela Venkata, Rajashekhar Mandla, Srinivas Akula, Mallaiah Bedika, Bhadru Dharavath, Sreelatha Dogga, Ramakrishna Babu A, Chandrasekhar Javaji

**Affiliations:** ^1^ Maize Research Centre, Agricultural Research Institute, Professor Jayashankar Telangana Agricultural University, Hyderabad, India; ^2^ Department of Entomology, College of Agriculture, Professor Jayashankar Telangana Agricultural University, Hyderabad, India; ^3^ Institute of Biotechnology, College of Agriculture, Professor Jayashankar Telangana Agricultural University, Hyderabad, India; ^4^ Market Intelligence Centre, College of Agriculture, Professor Jayashankar Telangana Agricultural University, Hyderabad, India; ^5^ All India Coordinated Research Project (AICRP) on Biological Control of Crop Pests and Diseases, Professor Jayashankar Telangana Agricultural University, Hyderabad, India; ^6^ Indian Council of Agriculture (ICAR)-Indian Institute of Maize Research, Winter Nursery Centre, Hyderabad, India

**Keywords:** fall armyworm, pheromone trap catches, climatological parameters, INGARCHX, ANNX, SVRX

## Abstract

The fall armyworm (*Spodoptera frugiperda*) poses a significant threat to global maize production owing to its rapid life cycle, extensive host range, and strong dispersal capabilities. We developed a forecasting system for fall armyworm outbreaks over one week using weekly pheromone trap counts (2019–2023) from the Maize Research Centre in Rajendranagar (Hyderabad), combined with weather data such as air temperature, relative humidity, and rainfall. Three modelling approaches, INGARCHX, SVRX and ANNX, were evaluated based on performance metrics: Integer Valued GARCH with Exogenous Variables (INGARCHX), Support Vector Regression with climate inputs (SVRX), and Artificial Neural Network with climate inputs (ANNX). During the training phase, the ANNX model delivered the best performance, recording a mean square error of 0.42 and a root mean square error of 0.65. These results outperformed the SVRX model, which produced a mean square error of 7.29 and a root mean square error of 2.70, and also exceeded the INGARCHX model, showing a mean square error of 2.91 and a root mean square error of 1.70. During testing, the ANNX model consistently outperformed the alternatives, yielding a mean squared error of 25.13 and a root mean squared error of 5.01. SVRX recorded scores of 34.07 and 5.84, while INGARCHX showed 48.90 and 6.99, respectively. Diebold–Mariano tests verified that ANNX’s edge over SVRX and INGARCHX is statistically significant at the 5%. By integrating climate variables, this neural network is a dependable early-warning system that predicts fall armyworm population surges with roughly 80% accuracy, one week ahead. This timely and geographically targeted forecasting allows for precise pest-control actions, minimizing maize yield losses and advancing sustainable agricultural strategies.

## Introduction

The fall armyworm (*Spodoptera frugiperda* J.E. Smith; Lepidoptera: Noctuidae) now ranks among the most damaging invasive pests worldwide, posing a serious threat to food security on a global scale. Notably, it has damaging effects on cereal crops like maize. Native to the Americas, FAW was first detected in India in 2018 and has since rapidly spread, infesting nearly 90% of maize-growing areas (Suby et al., 2020). Its high mobility, ability to fly up to 500 km with wind currents, and a wide range of over 350 plant species make it a serious pest (Montezano et al., 2018; CABI, 2020). FAW has been reported in more than 100 countries (Prasanna et al., 2018; Baloch et al., 2020), highlighting its potential to invade.

In India, FAW is seen as a high-priority pest. Although many integrated pest management (IPM) strategies have been created, chemical control is still the primary method used (Kalisetti et al., 2024). Climate change makes pest management harder by changing the interactions between pests, hosts, and the environment. Temperature, rainfall, and CO_2_ levels affect FAW’s reproduction, development, and movement (Prakash et al., 2014; Boggs, 2016). For instance, a 1.5 °C increase in temperature could lead to a 45-58% rise in the number of days over 35 °C. This may enlarge FAW’s habitat and increase generational turnover (IPCC, 2023; Ramírez-Cabral et al., 2020).

Given these challenges, it is important to understand how weather factors affect FAW population changes. This understanding is key for timely pest monitoring, predicting outbreaks, and taking preventive actions. Pheromone traps are commonly used to monitor FAW populations. They provide valuable information about seasonal activity and where FAW is found (Rajashekhar et al., 2024). However, while extensive modelling studies exist for FAW in Africa and the Americas, research tailored to Indian agroclimatic conditions remains scarce.

Forecasting pest populations has traditionally relied on statistical models such as multiple regression and ARIMA. Although useful, these models are limited when applied to non-Gaussian, count-based pest data, even with transformations for normality. Though Phenology and degree-day models are widely used and effective in pest modelling (including for fall armyworm), they also have notable limitations. While phenology and degree-day models help predict pest development, they have limitations. For example, they depend on temperature as the only factor driving development and may overlook important ecological elements like rainfall, changes in host plants, or pest movement. Additionally, linear DD models might oversimplify the complex biological responses to temperature extremes. These limitations can impact prediction accuracy in the fall armyworm, a highly migratory and polyphagous species.

Count time series models, such as INGARCH, designed for discrete, autocorrelated data, offer a more suitable alternative but remain underutilised in pest forecasting (O’Hara and Kotze, 2010; St-Pierre et al., 2018). Meanwhile, machine learning (ML) methods—particularly Support Vector Regression (SVR) and Artificial Neural Networks (ANN)—have shown strong predictive power in agriculture due to their ability to capture non-linear relationships without assuming data normality. These techniques have been used for crop yield forecasting (Amaratunga et al., 2020; Rathod et al., 2018), rice pest prediction (Ma et al., 2019), and sugarcane borer disease modelling (Huang et al., 2018). However, their application to FAW population forecasting remains limited, especially in an Indian context.

Count-based time series models and ML techniques have also been applied in diverse domains, including stock markets (Fokianos et al., 2009), manufacturing claims (Weiß, 2009), disease surveillance (Zhu and Wang, 2010; Tanawi et al., 2021), and network traffic analysis (Kim, 2020). In agriculture, Kim et al. (2014) and Alam et al. (2018) point out the possibilities of combining machine learning with time series data. However, there is no comparative evaluation of these methods for pest forecasting in India. Given the growing threat of FAW due to changing climate conditions and the limitations of current forecasting methods, there is a clear need for strong, location-specific models that combine climate data with improved forecasting techniques.

This study aims to fill this gap by:

- Examining seasonal trends in FAW populations using pheromone trap data.
- Identifying key meteorological variables that influence FAW dynamics in maize-growing regions.
- Developing predictive models using both:
  i. Count time series frameworks (e.g., INGARCH), and
  ii. Machine learning techniques (e.g., SVR and ANN),
  iii. To capture the non-linear and discrete nature of FAW count data.
- Comparing model performance to determine the most accurate and reliable approach for FAW forecasting.
- Supporting early warning systems for FAW through integrated, data-driven forecasting tools that inform IPM strategies and reduce yield loss.

## Materials and methods

### Study site and experimental design

**Figure 1:**
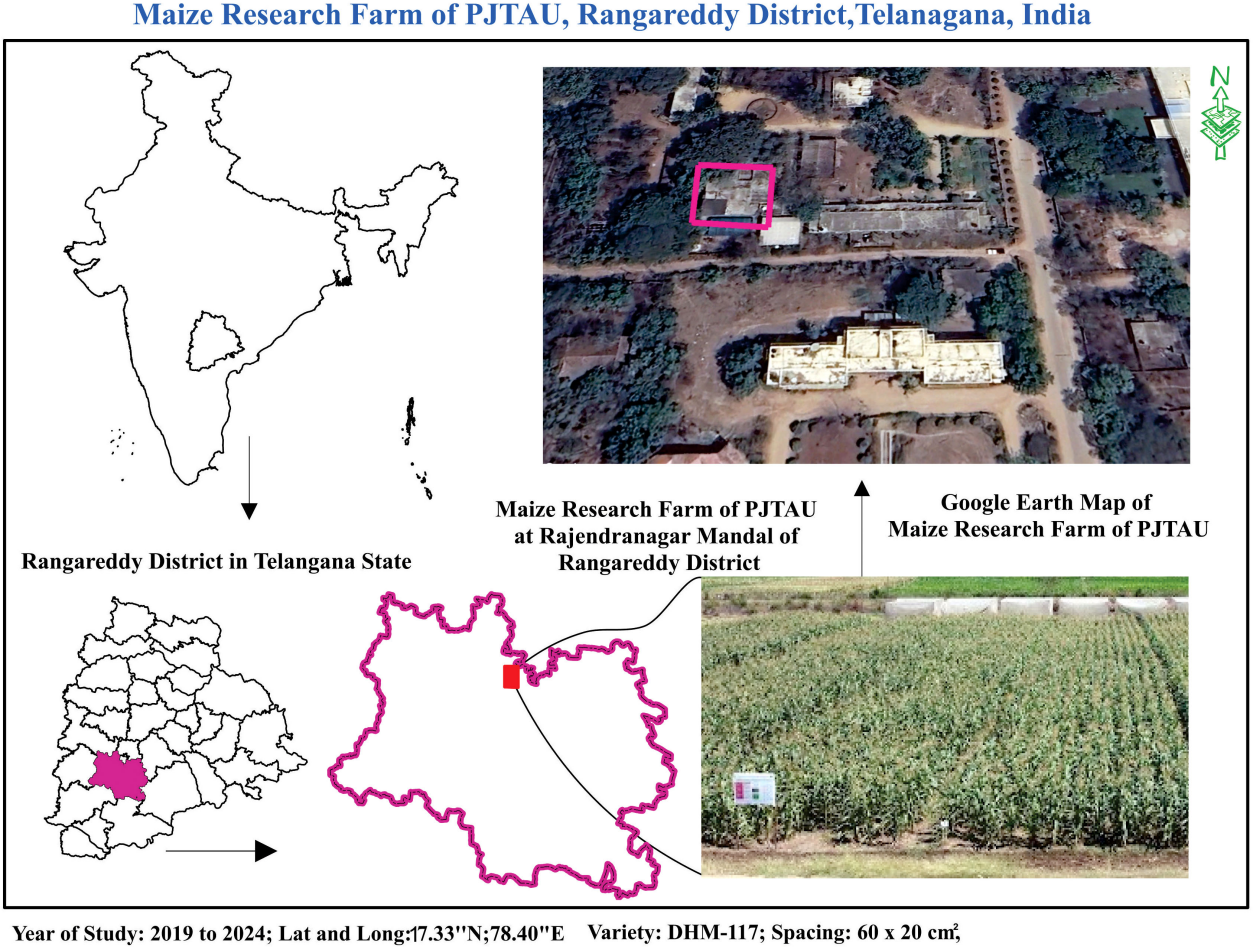
Location of the fall armyworm modelling study.

A fixed-plot field experiment (8000m²) took place over five years. It spanned ten consecutive cropping seasons: *Kharif* 2019, *Rabi* 2019-20, *Kharif* 2020, *Rabi* 2020-21, *Kharif* 2021, *Rabi* 2021-22, *Kharif* 2022, *Rabi* 2022-23, *Kharif* 2023, and Rabi 2023-24. The study was conducted at the Maize Research Centre, Rajendranagar, Hyderabad, Telangana, India (17.33°N, 78.40°E) (**Figure 1**) in the Southern Agro-Climatic Zone of Telangana. The region experiences a semi-arid tropical climate with an average annual temperature of ~22°C. The soil is sandy loam, and irrigation is available. Each season, two bulk plots of 4000 m² each were sown with maize hybrid DHM 117 using a spacing of 60 cm × 20 cm. Standard agronomic practices were followed uniformly, excluding pest control measures to ensure natural FAW incidence.

### Data collection

**Figure 2:**
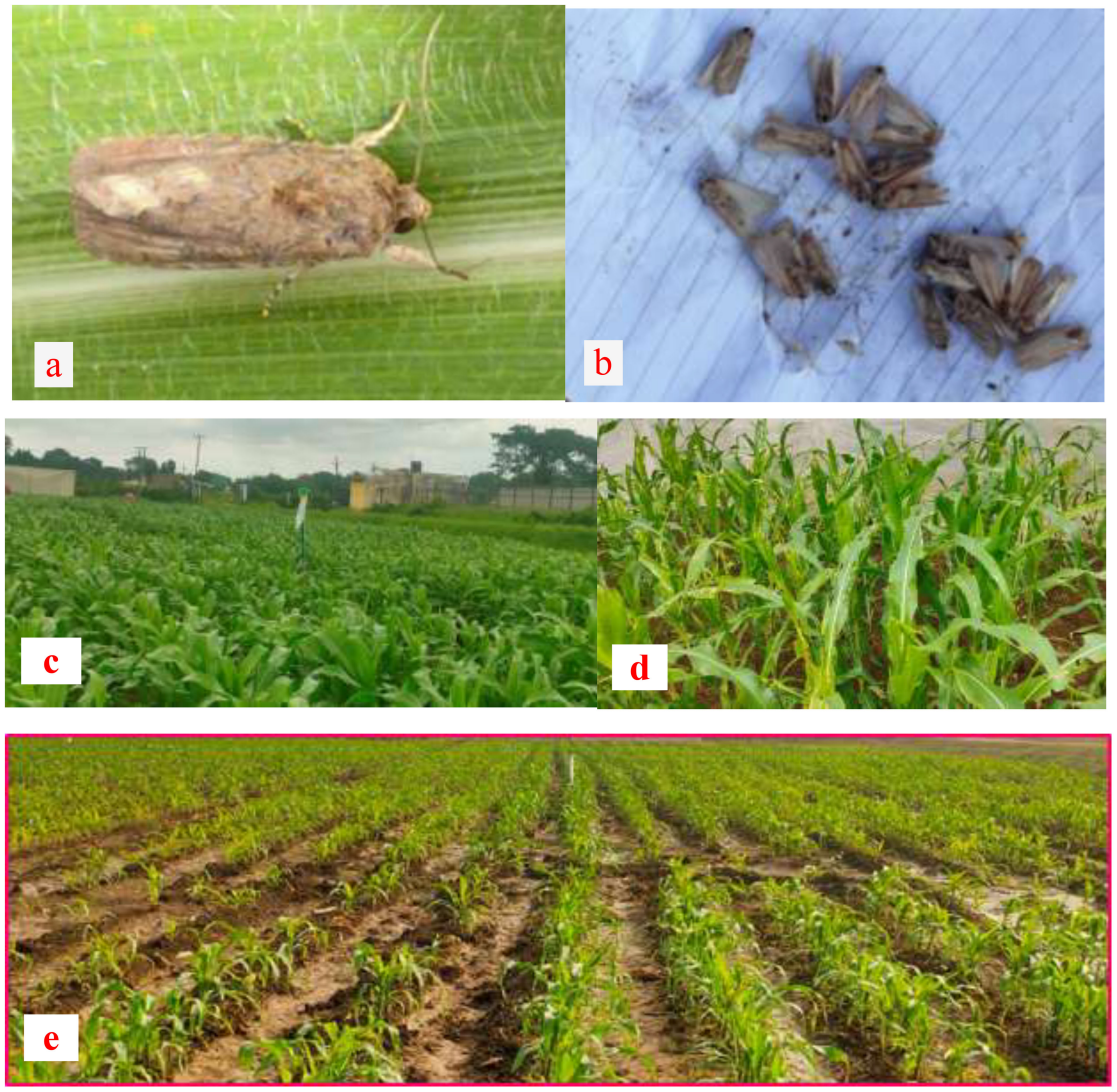
**(a)** Adult Fall armyworm, *Spodoptera frugiperda*. **(b)** Adult trap catches **(c)** Pheromone traps in the field. **(d)** Damage symptoms of FAW, **(e)** Field view of the Experimental trial.

FAW monitoring: Funnel traps with slow-release NBAIR pheromone lures were installed to monitor adult Spodoptera frugiperda (FAW). Trap installation began 7 days after crop germination (V2 stage) and continued until crop maturity. Lure replacement occurred every 4 weeks. Daily trap counts were recorded and later aggregated to weekly averages per trap. One trap was installed per 1000 m² plot, with a total of 8 traps covering two plots (8000 m²). Captured specimens were taken to the laboratory for manual counting and identification (**Figure 2**).

Weather data: Meteorological parameters included maximum temperature (MaxT), minimum temperature (MinT), morning relative humidity (RHM), evening relative humidity (RHE), and rainfall (RF). Weekly averages of these parameters were aligned with Standard Meteorological Weeks (SMW). Data were sourced from an automatic weather station at the Agro Climate Research Centre, Rajendranagar, Hyderabad.

Statistical analysis: Descriptive statistics including mean, standard error (SE), coefficient of variation (CV), skewness, kurtosis maximum and minimum were used to summarise FAW counts and meteorological data. Time series plots were created to visualise temporal trends. Pearson’s correlation was employed to assess relationships between FAW counts and weather variables. Stepwise multiple regression was used to identify key meteorological predictors of FAW populations, based on the model: Y=Xβ+e, e∼N(0,σ2)Y = X\beta + e, \quad e \sim N(0, \sigma^2), where Y represents the dependent variable (weekly FAW counts), X is the matrix of meteorological predictors, β\beta is the vector of regression coefficients, and e is the error term. Analyses were conducted using R software (R Core Team, 2018) for time series models and machine learning and SAS software version 9.3 (SAS, 2011) for correlation and regression analyses.

### Predictive modelling approaches

#### INGARCHX model (count time series)

The Integer-Valued Generalised Autoregressive Conditional Heteroscedastic (INGARCH) model is designed for count time series data. It models FAW trap counts using historical values and meteorological covariates. Poisson and Negative Binomial distributions were tested to handle over-dispersion (Kedem and Fokianos, 2002; Heinen, 2003; Ferland et al., 2006; Zhu, 2012). The INGARCHX model extends the traditional INGARCH by including exogenous variables (e.g., MaxT, MinT, RF, RHM, RHE).

Consider the “count time series denoted as (Yt: t ∈ N) and the time-varying r-dimensional covariate vector as (Xt: t ∈ N), where Xt = (Xt,1,…, Xt,r)^T^. E defines the conditional mean (Yt/Ft-1) = Yt, with Ft symbolizing the historical data. The generalised form of the model (Equation 1) is articulated as follows:


(1) $$g({\text{λ}}_{\text{t}})={\text{β}}_{0}+\sum_{\text{k}=1}^{\text{p}}\alpha_{\text{k}}\tilde{g}\;({\text{Y}}_{\text{t}-{\text{i}}_{\text{k}}})+\sum_{\text{l}=1}^{\text{q}}{\text{β}}_{1}g({\text{λ}}_{\text{t}-{\text{j}}_{1}})+{\text{η}}^{\text{T}}$$

Case 1: Imagine a situation where both g and ğ are identity functions, meaning g(x) = x and ğ(x) = x. Under these conditions, Yt adheres to a (Poisson) INGARCH (p, q) model (Equation 2) with p greater than one and” q greater than zero if the following hold true: (a) Yt, when “conditioned on Yt-1, Yt-2, and so on, follows a Poisson distribution. (b) The conditional mean λt =E[Yt | Yt-1, Yt-2,…] meets the criteria:


(2) $$\lambda_{t}=\beta_{0}+\sum_{i=1}^{p}\alpha y_{t-i}+\sum_{j=1}^{q}\beta_{j}\lambda_{t-j}\text{ with }\beta_{0\;}>0\text{ and }\alpha 1,\ldots ,\alpha p,\ldots ,\beta_{1},\ldots ,\beta_{q}\geq 0$$

This leads to an INGARCH order p and q model called the INGARCH (p, q)” model, assuming that Yt | Yt-1 has a Poisson distribution. The INAGARCH (p) model is (Equation 3) employed when q equals 0 (Fokianos et al., 2009). These models are sometimes referred to as “ACP (Autoregressive Conditional Poisson)” models.

Case 2: The conditional variance might exceed the mean λt in the negative binomial distribution; this is known as over-dispersion and is determined by the over-dispersion parameter Ø (Christou and Fokianos, 2014). Yt | Ft-1 is assumed to follow NegBinom (λt, Ø), a Negative Binomial distribution. As Ø tends toward infinity, the Poisson distribution is a limiting case of the negative binomial distribution under this premise.


(3) $${\text{Y}}_{\text{t}}|{\text{Y}}_{\text{t}-1},{\text{ Y}}_{\text{t}-2},\ldots ,|\sim \text{Bin}(\text{n},\text{β}+{\text{α Y}}_{\text{t}-1/\text{n}})$$

Further insights into estimating INGARCH models through conditional” likelihood estimation, with an emphasis on asymptotic properties, are available in Heinen (2003) and Fokianos (2011). Assuming that future values are impacted by the target variable’s past values and the prior values of exogenous variables, the traditional INGARCH model forecasts future values exclusively based on the target variable’s historical values. By incorporating additional exogenous factors into the prediction model, the INGARCHX model extends this further (Liboschik et al., 2020).

### Support Vector Regression

Support Vector Regression (SVR) maps input data into a high-dimensional feature space using kernel functions, most commonly the Radial Basis Function (RBF). Its objective is to minimise a regularised risk function, striking a balance between model complexity and prediction error. The performance of SVR largely depends on key hyperparameters, particularly C, which controls the regularisation strength, and γ, which defines the kernel bandwidth.

SVR models incorporated meteorological variables as exogenous predictors of FAW counts. In order to create the “regression or time series model, SVR maps the original input space into a high-dimensional feature space (Vapnik, 1995). A dataset is represented as Z = {xi yi}^N^i=1, where xi ϵ R^n^ represent the input vector, yi represents the scalar output, and N represents the dataset size. The general equation for SVR (Equation 4) can be expressed as follows:


(4) $$f(x)=w^{T}\phi (x)+b$$

In this context, W signifies the weight vector, b is the bias term, and the superscript T denotes the transpose. Coefficients W and b are derived from the data by minimising the subsequent regularised risk function (Equation 5):


(5) $$R(\theta )=\frac{1}{2}∥w{∥}^{2}+c[\frac{1}{N}\Sigma_{i=1}^{N}L_{\epsilon }(y_{i},f(x_{i}))]$$

This regularised risk function helps avoid underfitting and overfitting the model by concurrently minimising the regularisation term and the empirical error. The first term in Equation 5, $\frac{1}{2}∥w$ ^2^, is known as the “regularisation term.” It quantifies how flat the function is. The function is advised to be as “flat as possible by minimising $\frac{1}{2}∥w∥$ ^2^. The second term, $1\frac{1}{N}\;\sum_{i=1}^{N}L\in (yi,f(xi))$ is called the ‘empirical error,’ that is estimated by employing Vapnik’s e-insensitive loss function (Equation 6), as follows:


(6) $$L_{\epsilon }(y_{i},f(x_{i}))=f(x)=\{\begin{array}{ll} \left|y_{i},f(x_{i})-\epsilon \right|; & \left|y_{i}-f(x_{i})\right|\geq \epsilon ,\\ 0 & \left|y_{i}-f(x_{i})\right|<\epsilon , \end{array}$$

$Y_{i}$ represents the actual value, and f ( $x_{i}$ ) is the estimated value. The “RBF (Radial Basis Function)” is the most frequently employed kernel function (Equation 7), expressed as follows:


(7) $$k(x_{i},x_{j})=\;exp\{-\gamma ∥x-x_{i}{∥}^{2}\}$$

**Figure 3:**
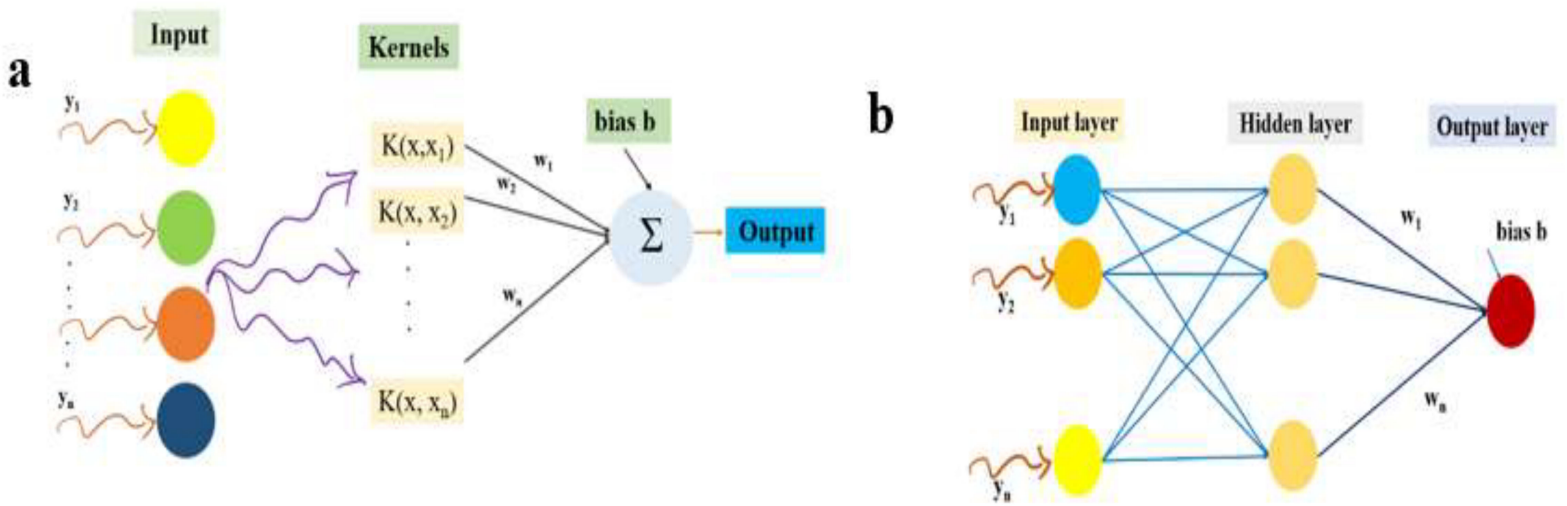
SVR **(a)** and ANN **(b)** model architectures.

The architecture of the SVR is shown in **Figure 3**.

### Artificial Neural Network

Artificial Neural Network with Exogenous Inputs (ANNX) is a multi-layer feedforward ANN architecture that was implemented with past pest counts and meteorological variables as the input layer, an optimised number of neurons in the hidden layer, and predicted FAW counts in the output layer. The ANN model captures complex non-linear relationships through iterative weight updates during training. Over recent decades, ANNs have become one of the most widely employed machine learning methods. In time series modelling, they are often referred to as autoregressive neural networks because they rely on time-lagged inputs. A neural network that natively models the temporal function can quantitatively represent the time series method for an ANN. The following is the expression for a multi-layer feedforward autoregressive neural network’s final output (Yt) (Equation 8).


(8) $$Y_{t}=\alpha_{0}+\sum_{j=1}^{q}\alpha_{j}g(\beta_{0j}+\sum_{i=1}^{p}\beta_{ij}Y_{t-p})\;+\epsilon_{t}$$

Here, $\alpha_{j}$ (j = 0, 1, 2,., q) and $\beta_{ij\;}$ (i = 0, 1, 2,…, p, j = 0, 1, 2,…, q) represent the model parameters, also known as the synopsis weights. The activation function is denoted by g, the number of input nodes by p, and the number of hidden nodes by q. An ANN’s training phase aims to reduce the error function between the predicted and actual values. An autoregressive ANN’s error function (Equation 9) is specified as follows:


(9) $$E=\frac{1}{N}\Sigma_{t=1}^{N}{\left(e_{t}\right)}^{2}=\frac{1}{N}\Sigma_{t=1}^{N}{\left\{\begin{array}{c} X_{t}- \end{array}(w_{0}+(\sum_{J=1}^{Q}w_{J}g(w_{o_{j}}+\Sigma_{\text{i}=1}^{p}w_{ij}X_{t-i})))\right\}}^{2}$$

Where “N is the total number of error terms. The parameters of the neural network $w_{ij}$ are adjusted by a change in as, $\Delta w_{ij}$ as $\Delta w_{ij}=-n\frac{\partial E}{\partial w_{ij}}$ , where n is the learning rate (Rathod and Mishra, 2018 and Zhang, 2003). The ANNX model will be formed by modelling the pest count using an exogenous variable, similar to the INGARCHX and SVRX models. The” ANN architecture is shown graphically in **Figure 3**.

For evaluating model performance, “MSE (Mean Square Error)” and “RMSE (Root Mean Square Error)” were used as comparison criteria. The MSE (Equation 10) is calculated as the average of the sum of squared error values and is expressed as:


(10) $$MSE=\frac{\sum_{i=1}^{N}{\left(y_{i}-{\hat{y}}_{i}\right)}^{2}}{N}$$

In regression analysis, RMSE (Equation 11) is also referred to as the standard error of the estimate and is defined as follows:


(11) $$RMSE={\sqrt{\frac{\sum_{i=1}^{N}(y_{i}-{\hat{Y}}_{i})}{N}}}^{2}$$

Here, $Y_{i}$ represents the actual value, ${\hat{y}}_{i}\;$ signifies the predicted value, and N denotes the number of observations

Diebold and Mariano invented the “Diebold–Mariano (DM)” test in 1995. It compares the residuals of models to see whether variations in predictive accuracy are statistically significant. Let d_i_ stand for the absolute difference between the residuals of the two competing models, r_1_ and r_2_.

d_i_ = |r_1_| - |r_2_|, and the auto covariance function γk (Equation 12) is defined as:


(12) $$\gamma_{k}=\frac{1}{n}\sum_{i=k+1}^{n}(d_{1}-\bar{d})(d_{i-k}-\bar{d})$$

The DM test statistic (Equation 13) is formulated as:


(13) $$DM=\frac{\bar{d}}{\sqrt{[\gamma_{0}+2\sum_{k=1}^{h-1}\gamma_{k}]/n}}$$

Where, h = n^1/3^ + 1. For hypothesis testing, the null hypothesis (H_0_) and the alternative hypothesis (H_1_) are defined as follows: H_0_ = E(d) = 0, indicating that the forecast accuracy is similar for both models, and H_1_ ≠ E(d) ≠ 0, suggesting that the forecast accuracy differs between the two models.

This study integrates climatological data with advanced statistical and machine learning models to forecast FAW populations in maize ecosystems of southern India. Three modelling approaches, INGARCHX, SVRX, and ANNX, are compared using standardised evaluation metrics. This supports the development of an early warning system for sustainable pest management.

## Results

**Figure 4:**
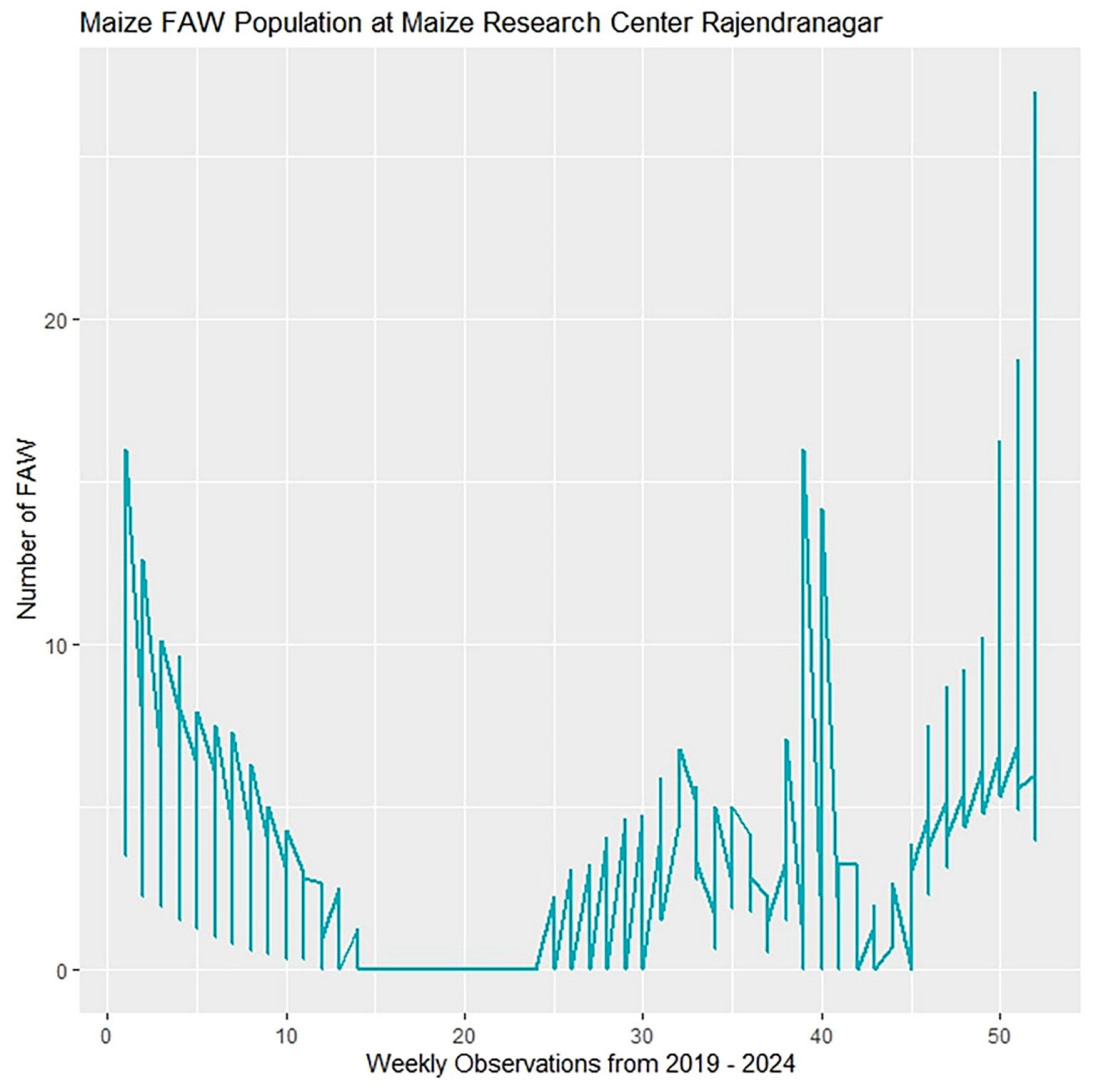
Fall armyworm populations of time series from 2019–2024.

**Figure 4** shows time series plots of weekly counts, by Standard Meteorological Week (SMW), of fall armyworm pheromone trap catches at the study site from 2019 to 2024. The graph reveals a higher incidence of fall armyworm (FAW) during the 52^nd^ SMW, with notable peaks around the 39^th^ and 52^nd^ SMWs.

**Figure 5:**
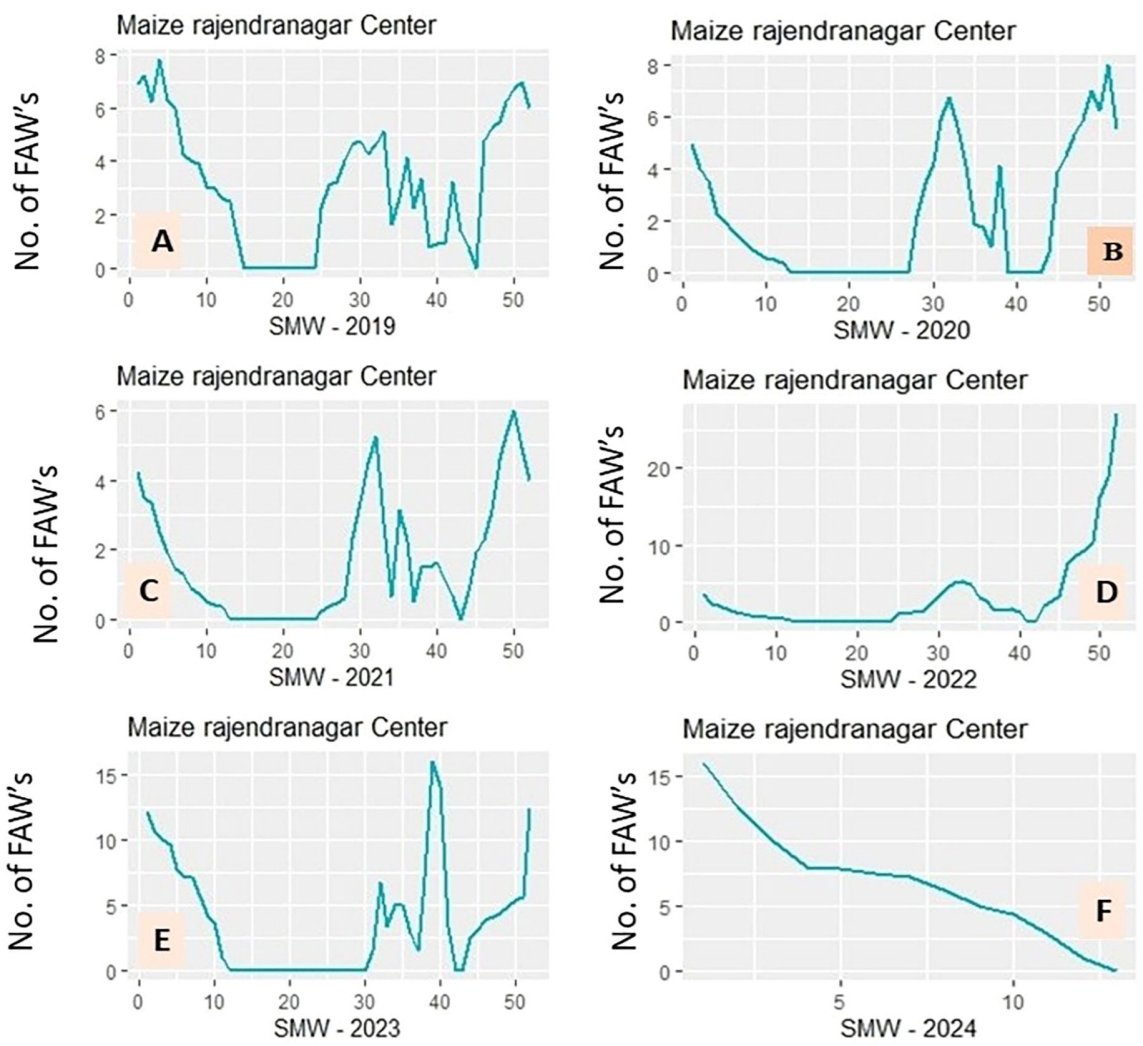
**(A-F)** Year-wise fall armyworm populations from 2019–2024.

**Figures 5A-F** display annual time series plots of FAW catches, illustrating year-to-year variation in population dynamics at the study site. FAW incidence exhibited distinct seasonal peaks: peaks occurred during the 4^th^ SMW in 2019, during the 51^st^ SMWs; in 2020, during the 33^rd^ and 51^st^ SMWs; in 2021, during the 32^nd^ and 50^th^ SMWs; in 2022, during the 51^st^ SMW; and in 2023, during the 39^th^ SMW. The 1^st^ SMW had the largest FAW infestation in 2024. High incidence levels continued into June.

### Descriptive statistics of FAW and weather variables

**Table 1** displays summary statistics for the dependent variable, the FAW population, and the exogenous climatic conditions. The FAW population shows high variability, ranging from 0 to 27 individuals per trap, and a strong positive skewness (2.339), indicating that while most trap catches were low, there were occasional large infestations. Rainfall showed the highest variability, with a coefficient of variation of 190.29%. It had extreme values and a strong positive skew of 2.658. This suggests it can significantly trigger pest incidence. Morning relative humidity was fairly consistent but negatively skewed at -1.191. This indicates that high humidity levels were common during this time. Temperature variables were moderately stable. The maximum temperature showed a slight positive skew of 0.673, while the minimum temperature had a slight negative skew of -0.496. Overall, the weather parameters displayed various patterns and levels of variability. They likely have a significant impact on FAW population dynamics.

**Table 1:** Summary statistics of fall armyworm pheromone-trapped individual collections at Maize Research Centre, Hyderabad.

Statistics	FAW population	Max T (°C)	Min T (°C)	RHM (%)	RHE (%)	Rainfall (mm)
Mean	2.939	32.184	19.335	86.251	52.360	19.683
SE.	0.221	0.225	0.245	0.483	0.984	2.267
Skewness	2.339	0.673	-0.496	-1.191	0.186	2.658
Kurtosis	8.880	-0.549	-0.278	1.973	-0.733	7.334
Minimum	0.000	24.710	5.500	51.570	17.430	0.000
Maximum	27.000	42.000	27.570	98.860	92.570	202.000
CV (%)	124.008	11.528	20.896	9.260	31.053	190.293

### Correlation analysis between FAW and meteorological variables

The Pearson correlation coefficients between the study’s climate factors and FAW populations are in **Table 2**. The fall armyworm (FAW) population had significant negative correlations with both maximum temperature (r = -0.440) and minimum temperature (r = -0.453). Higher temperatures are likely linked to lower trap catches because extreme temperatures may reduce pest activity or survival. Morning relative humidity (RHM) showed a weak but significant positive correlation with FAW (r = 0.158). This suggests that higher morning humidity slightly supports pest presence. In contrast, evening relative humidity (RHE) showed a weak negative correlation (r = -0.139), indicating a minimal inverse relationship. Rainfall also negatively correlated with FAW (r = -0.164), implying that increased rainfall may limit adult moth activity or cause larval mortality within the maize whorls, resulting in decreased trap captures.

**Table 2:** Coefficients of the Pearson correlation between meteorological variables and fall armyworm pheromone trap collections.

	FAW	MaxT	MinT	RHM	RHE
MaxT	-.440^**^				
Min T	-.453^**^	.419^**^			
RHM	.158^**^	-.650^**^	-.058		
RHE	-.139^*^	-.388^**^	.437^**^	.645^**^	
Rainfall	-.164^**^	-.272^**^	.274^**^	.367^**^	.615^**^

* indicates significant at 5% and ** indicates significant at 1%.

Overall, the correlation results indicate that FAW activity is adversely affected by higher temperatures and rainfall, while morning humidity has a slight favourable effect. The meteorological variables are also interrelated, especially regarding temperature and moisture, which likely contribute to the complexity of FAW population dynamics.

### Stepwise regression analysis of FAW trap catches and climatic variables

The climatological parameters affecting the growth of FAW populations were identified using a stepwise regression analysis. The findings are summarised in **Table 3**. The stepwise regression model found that maximum temperature (MaxT), rainfall (RF), and evening relative humidity (RHE) significantly predict fall armyworm (FAW) pheromone trap collections. The model’s intercept was 24.99 (SE = 2.07), which estimates the FAW population when all predictor variables are zero. Maximum temperature significantly negatively impacted FAW trap catches, with a coefficient of -0.577 (SE = 0.054), an F-value of 52.83, and a p-value of 0.00002. This factor accounted for 28.1% (R² = 0.281) of the variation. Rainfall also had a negative influence, with a coefficient of -0.015 (SE = 0.006), an F-value of 41.12, and a p-value of 0.0021, contributing to a cumulative R² of 0.314. Evening relative humidity (RHE) was the last variable included. It had a coefficient of -0.061 (SE = 0.016), an F-value of 32.58, and a p-value of 0.0007. This raised the model’s explanatory power to a total R² of 0.327. These results indicate that unfavourable weather conditions, particularly higher temperatures, rainfall, and evening humidity, negatively influence FAW trap catches, collectively explaining 32.7% of the variation observed.

**Table 3:** Stepwise Regression study of fall armyworm pheromone trap collections and climatological variables.

Centre	Variable	Estimate	SE.	F-value	Pr>F	R^2^	Model R^2^
Maize Research Centre Hyderabad	Intercept	24.99	2.07	25.96	0.01	–	-
	MaxT	-0.577	0.054	52.83	0.00002	0.281	0.327
	RF	-0.015	0.006	41.12	0.0021	0.314	-
	RHE	-0.061	0.016	32.58	0.0007	0.327	-

The regression results reveal that all three climatological variables—maximum temperature, rainfall, and evening relative humidity—significantly negatively affect FAW trap catches. The model explains approximately one-third (32.7%) of the variability in the FAW population, highlighting how bad weather conditions, like higher temperatures, rainfall, and humidity, affect FAW activity’s decline and the effectiveness of the traps.

### INGARCHX model assessment for fall armyworm populations

The INGARCHX (Integer-valued Generalised Autoregressive Conditional Heteroscedasticity with Exogenous variables) model was applied to assess the relationship between fall armyworm (FAW) trap counts and various weather parameters, as shown in **Table 4**. The intercept estimate was tiny (2.03 × 10^−^³) with a significant standard error (1.63792), and it was not statistically significant (Z = 0.0012, p = 0.9990), indicating that the intercept had minimal influence on the model. The autoregressive parameter β_1_, however, was highly significant (estimate = 0.76248, SE = 0.0988, Z = 7.7178, p = 0.0001), suggesting that current FAW populations were strongly dependent on their previous values, highlighting the importance of temporal autocorrelation in FAW population dynamics. In contrast, all meteorological variables—including maximum temperature, minimum temperature, morning and evening relative humidity, and rainfall—had negligible coefficient estimates and were statistically non-significant (p-values ranging from 0.7180 to 1.0000), indicating that within the INGARCHX framework these factors did not contribute significantly to explaining variation in FAW counts once temporal effects were accounted for. The model also revealed an overdispersion parameter of 6.50, suggesting considerable variability beyond what would be expected in a standard Poisson distribution, thereby supporting an INGARCH-type model. The Box-Pierce test indicated strong autocorrelation in the original FAW time series (λ² = 202.3, p < 0.0001). At the same time, the residuals from the fitted model showed no significant autocorrelation (λ² = 4.0607, p = 0.04389), confirming that the INGARCHX model effectively captured the underlying time-dependent structure in the data.

**Table 4:** Assessment of INGARCHX model parameters for fall armyworm populations.

Parameters	Estimate	SE.	Z Value	p	Box-pierce non-correlation test	
					Original	Residuals
Intercept	2.03 x 10^-3^	1.63792	0.0012	0.9990	λ^2^ = 202.3 p-value≤ 0.0001	λ^2^ = 4.0607, p-value= 0.04389
beta_1	7.6248 x 10^-1^	9.8795 x 10^-2^	7.7178	0.0001184		
MaxT	4.2093 x 10^-8^	3.1768 x 10^-2^	0.0000	1.0000		
MinT	2.0947 x 10^-13^	2.7598 x 10^-2^	0.0000	1.0000		
RHM	2.5534 x 10^-5^	2.4463 x 10^-3^	0.0104	0.9917		
RHE	4.2676 x 10^-3^	1.1818 x 10^-2^	0.3611	0.7180		
Rainfall	2.9874 x 10^-5^	7.7692 x10^-3^	0.0038	0.9969		
Over dispersion Parameter	6.50					

SE, standard error; p, probability; λ^2^, chi-square test statistic.

The INGARCHX model revealed that their previous counts (autoregression) strongly influence FAW population levels. At the same time, weather variables did not show a significant direct impact in this time-series model. The model effectively accounted for autocorrelation and overdispersion, making it suitable for capturing the temporal dynamics of FAW populations.

### Comparison of SVRX and ANNX models for FAW population prediction

#### SVRX model

The parameters given in **Table 5** were used to create a “non-linear SVR model with exogenous variables for the fall armyworm population count time series. The SVRX model, which uses Support Vector Regression, employed a Radial Basis Function (RBF) as its kernel function with gamma = 0.2, a cost parameter of 1, and epsilon = 0.1, allowing for some tolerance in prediction error. The model utilised 186 support vectors and produced a cross-validation error of 0.213, indicating good generalisation performance. The Box-Pierce test for residuals in the SVRX model showed a λ² value of 134.03 with p < 0.001, suggesting significant autocorrelation remained in the residuals and that the model may not have fully captured the time-dependent structure of the data.

**Table 5:** Details of the ANNX and SVRX models’ parameters for fall armyworm populations. .

SVRX		ANNX	
Kernel function	RBF	Input lag	7
No. of Support Vectors	186	Dependent/output variable	1
Cost	1	Hidden layer	1
Gamma	0.2	Hidden nodes	4
Epsilon	0.1	Exogenous variables	5
Cross-validation error	0.213	Model	NNAR(7,4)
Box-Pierce non-correlation test for residuals	λ^2^ = 134.03 p-value≤ 0.001	Total number of parameters	57
		Network type	Feed Forward
		Activation function I: H	Sigmoidal
		Activation function H::O	Identity
		Box-Pierce non-correlation test for residuals	λ^2^ = 2.8024 p-value= 0.09412

I:H, Input to Hidden layer; H O, Hidden to Output layer.

#### ANNX model

The parameters of the ANNX model are shown in **Table 5**. Unlike SVRX, the ANNX model was created as a Feed Forward Neural Network using the NNAR (7,4) structure. This structure includes seven input lags, one hidden layer, and four hidden nodes. The model had five external variables and a total of 57 parameters. The activation function between the input and hidden layers was sigmoidal, while the output layer used an identity function. The Box-Pierce test for the ANNX model produced a λ² value of 2.8024 with a p-value of 0.09412. This result shows that the residuals were not significantly autocorrelated and that the model effectively captured the temporal structure in the data.

Both models were designed to consider outside factors when predicting FAW populations. The ANNX model showed better performance in managing time series dependencies, as indicated by its absence of significant residual autocorrelation. The SVRX model was effective in reducing prediction error but exhibited residual autocorrelation. This suggests that it was not as effective in modelling the time patterns of FAW dynamics. The ANNX model’s flexible structure and capacity to capture non-linear relationships make it a stronger choice for forecasting FAW populations.

### Model performance comparison on training and testing sets

The performance of three models, INGARCHX, ANNX, and SVRX, in predicting the occurrence of FAW is compared in **Table 6**. In the training dataset, the Artificial Neural Network with Exogenous variables (ANNX) performed best. It achieved the lowest Mean Squared Error (MSE = 0.42) and Root Mean Squared Error (RMSE = 0.65). This shows its high accuracy and good fit to the observed values. The INGARCHX model showed moderate performance, with an MSE of 2.91 and an RMSE of 1.70. In contrast, the Support Vector Regression with Exogenous variables (SVRX) had the highest training errors, with an MSE of 7.29 and an RMSE of 2.70. This indicates it was the least accurate during the training phase.

**Table 6:** Model comparison criteria for fall armyworm populations in training and testing datasets.

Location		Criteria	INGARCHX	SVRX	ANNX
Maize Research Centre, Rajendranagar Hyderabad	Training Set	MSE	2.91	7.29	0.42
		RMSE	1.70	2.70	0.65
	Testing Set	MSE	48.90	34.07	25.13
		RMSE	6.99	5.84	5.01

MSE, Mean Square Error; RMSE, Root Mean Square Error.

In the testing dataset, which checks how well the models generalize, the ANNX model again outperformed the others. It recorded the lowest MSE at 25.13 and an RMSE of 5.01. The SVRX model followed with an MSE of 34.07 and RMSE of 5.84, while the INGARCHX model showed the poorest performance on unseen data, with a significantly higher MSE of 48.90 and RMSE of 6.99.

The ANNX model was the strongest and most precise for predicting FAW populations across training and testing datasets. Its lower error values show that it learned patterns better and generalised to new data more effectively than SVRX and INGARCHX. While INGARCHX effectively captured time-based relationships in earlier analysis, its predictive accuracy was relatively low, particularly during testing. These results highlight how well neural networks can model complex, non-linear biological systems like FAW population dynamics.

## Discussion

The comparison of various models for predicting fall armyworm populations at the Maize Research Centre, Rajendranagar, Hyderabad, is detailed in **Table 6**, with a focus on MSE and RMSE for both training and testing datasets. The low R^2^ value of the stepwise regression model in this study indicates a poor fit, which is probably caused by the dependent variable’s high heterogeneity and nonlinearity. However, similar studies reported by Rathod et al. (2022) found a link between temperature, rainfall, and relative humidity and the growth of gall midge in rice over multiple generations.

**Figure 6:**
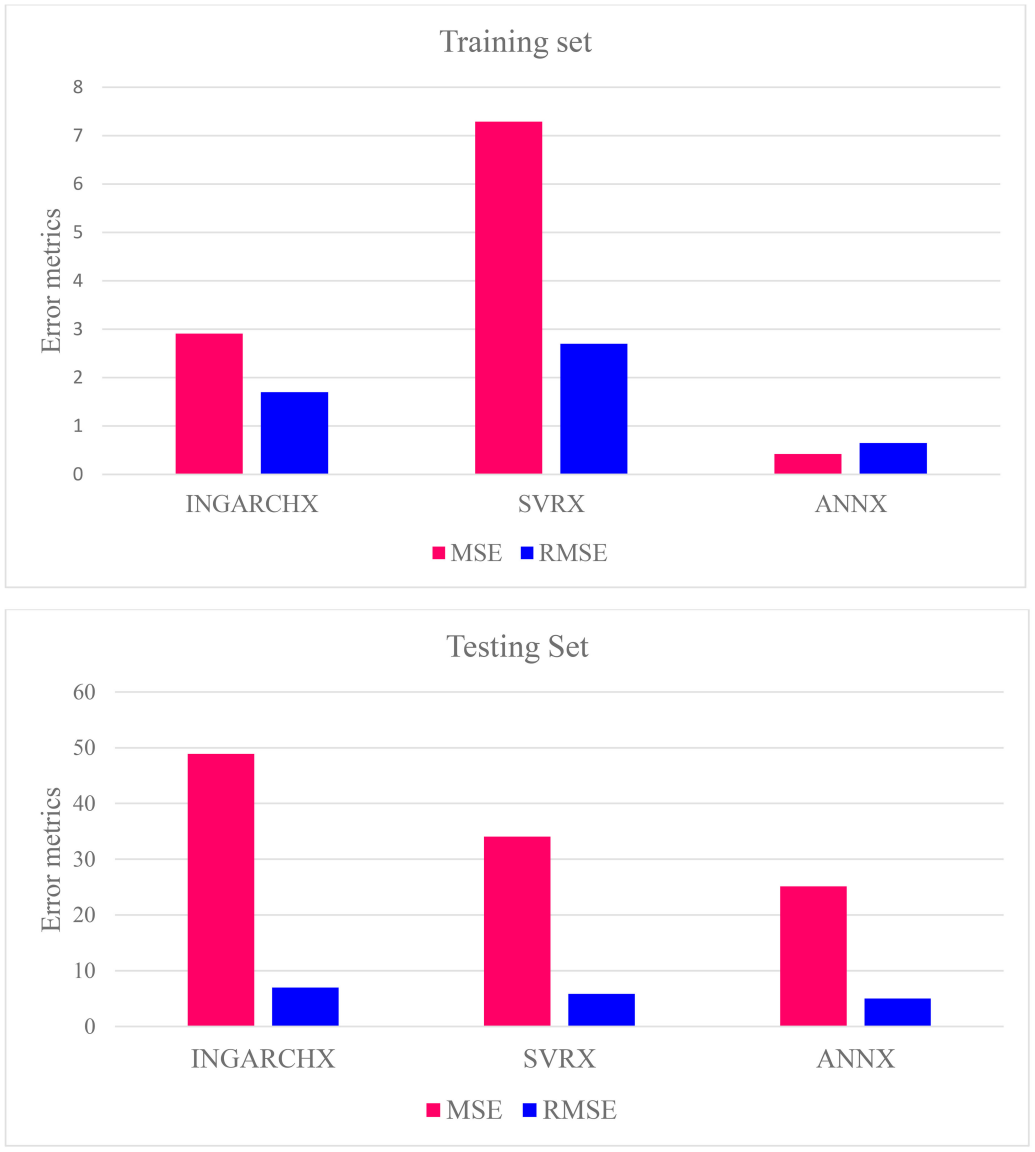
Comparison of performance of each model based on error metrics, MSE and RMSE of both testing and training sets.

The ANNX model fared better than the SVRX and INGARCHX models among the models tested in terms of RMSE and MSE for both the testing and training datasets (**Figure 6**). Furthermore, the SVRX model performed exceptionally well on the testing datasets. Still, the INGARCHX model outperformed it on the training datasets. The performance rankings of the models for training and testing datasets are ANNX, INGARCHX, and SVRX. These results match findings from related studies, like Rathod et al. (2022), where the ANN model performed better than traditional ARIMA and SVR models in predicting rice gall midge populations.

Each user-defined setting combination of SVR model hyperparameters was ten-fold cross-validated. **Table 5** displays the cross-validation error with the lowest value for each combination. Hyperparameter optimisation involved testing different combinations to identify the optimal parameters, striving to minimize training error while maintaining an acceptable error margin (epsilon). For the Artificial Neural Network model, the ‘Levenberg-Marquardt backpropagation algorithm’ was employed in a feedforward network, with multiple assessment rounds. We trained the network 25 times with a maximum of 1,000 iterations at a 0.03 learning rate and 0.01 momentum. Various hidden node designs and input lag values were investigated to reduce training mistakes, and model parameters were selected.

**Figure 7:**
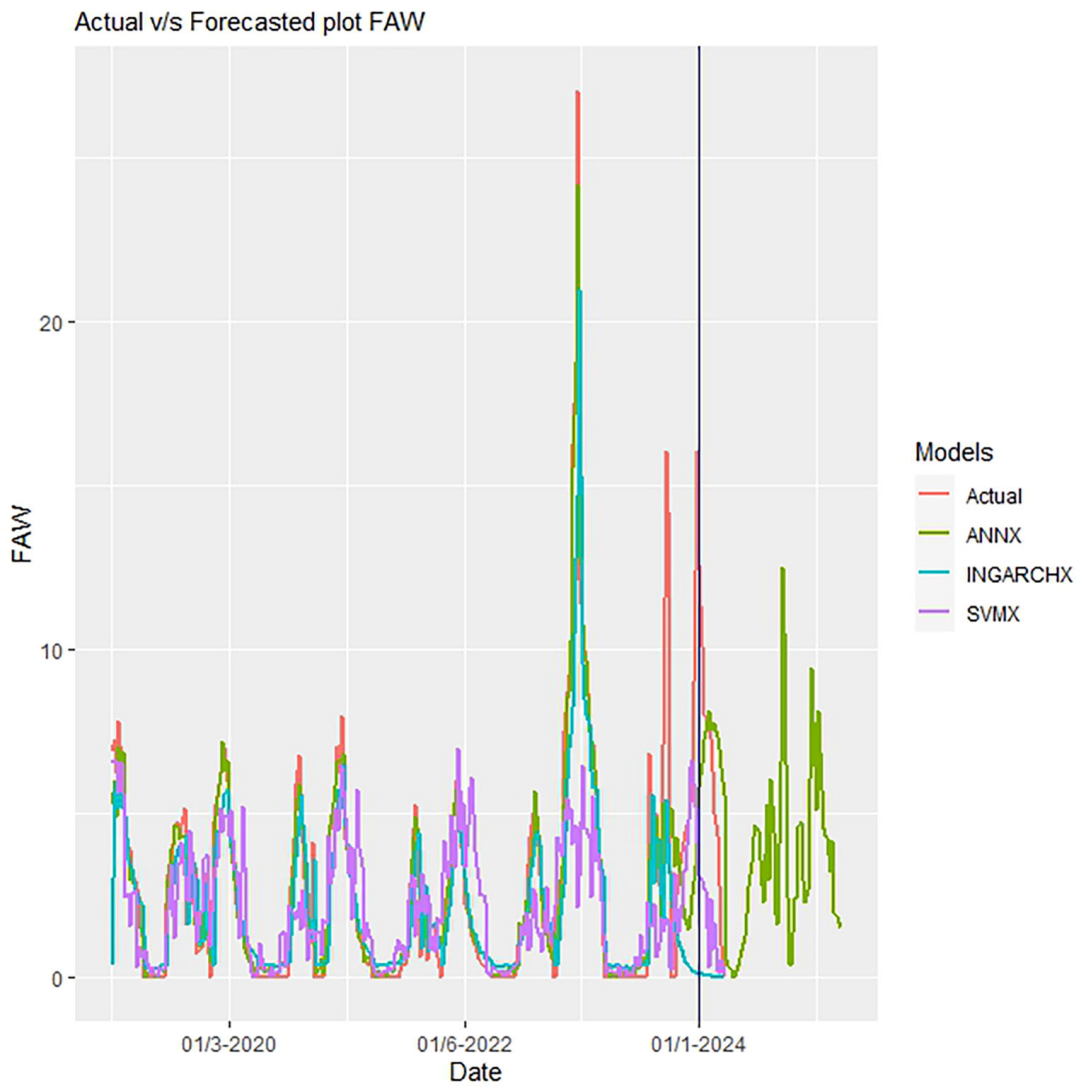
Actual vs. predicted plots of fall armyworm population.

The ANNX model’s prediction of the fall armyworm population was more precise than those of the INGARCHX and SVRX models (**Figure 7**). The differences between the models’ anticipated values are highlighted using metrics such as MSE and RMSE. The DM test statistic assessed significant statistical differences across the models. The findings supported the higher performance of the ANNX model by showing notable differences between the INGARCHX (M1) and SVRX (M2) models and the ANNX (M3) model (**Table 7**).

**Table 7:** Assessing model accuracy with the Diebold–Mariano Test: insights from Maize Research Centre, Hyderabad.

	Data type	M1, M2	M1, M3	M2, M3
Maize Research Centre, Rajendranagar, Hyderabad	Training	3.1758 (0.0017)	-2.5802 (0.0105)	-2.2625 (0.0323)
	Testing	-2.1229 (0.0348)	-6.087 (<0.0001)	3.6281 (0.0012)

M1: INGARCHX, M2: SVRX, M3: ANNX.

While the Artificial Neural Network model employs a Sigmoid-based activation function for mapping inputs to the hidden layer, “the RBF kernel function in SVR approaches a Gaussian distribution as the gamma value increases. This character may help to explain why the INGARCH model has trouble finding patterns in count time series data, which frequently come from non-Gaussian distributions. In assessing and forecasting rice gall midge population trends, ANN fared better than INGARCH and SVR, according to similar findings published by (Weiß, 2009). Because the ANNX model’s residuals were random and uncorrelated rather than non-random and correlated like those of” the SVRX and INGARCHX models, diagnostic evaluations further prove the ANNX model’s higher accuracy. The significant inter-model discrepancies are briefly outlined in **Table 7**.

Machine learning algorithms generally demonstrate stronger predictive performance, as supported by comparable studies: (Piekutowska et al., 2021) in early potato yield prediction (Liu et al., 2021, in projecting rice blast occurrences, and Haider et al., 2019 in forecasting wheat production in Pakistan. The ANNX model has demonstrated more precise predictions for fall armyworm outbreaks in field-level applications. This model provides farmers with important insights into how climate changes affect pest risk levels by using important weather variables, such as rainfall, minimum temperature, and relative humidity. Specifically trained on data from the Maize Research Centre in Rajendranagar, Hyderabad, the model is optimised for local predictions, which improves its usefulness for site-specific pest management.

The Artificial Neural Network with Exogenous variables (ANNX) model performed better than INGARCHX and SVRX. This is due to its flexibility in modelling non-linear relationships and its ability to capture complex patterns, like seasonality and time-related dependencies. Unlike traditional statistical models, neural networks are driven by data and do not depend on strict distribution assumptions. This allows them to respond more effectively to biological phenomena’ unpredictable and changing behaviour, such as fall armyworm (FAW) infestations. The NNAR (7,4) structure enabled the model to integrate lagged inputs and exogenous weather variables, capturing delayed responses and cumulative environmental effects that influence FAW populations.

ANN models, particularly those that use Levenberg-Marquardt backpropagation, are effective for learning non-linear mappings through repeated optimisation. A sigmoid activation function in the hidden layers allowed the ANNX model to manage non-Gaussian, skewed count data. This feature is typical of pest trap series (Weiß, 2009; Liboschik et al., 2020). Additionally, the random and uncorrelated residuals from the ANNX model show better model specification and less autocorrelation. This confirms its statistical validity, as shown in **Figure 7**.

The INGARCHX model, although suitable for count data and designed to handle overdispersion and autocorrelation, is constrained by its underlying Poisson or negative binomial assumptions, which may not hold for highly variable biological data like FAW counts. Moreover, its linear formulation limits its ability to detect complex non-linear interactions between weather variables and pest emergence. This limitation was evident in the model’s higher error values on the testing dataset, indicating weaker generalisation capability.

The SVRX model can model nonlinearity using kernel functions, but it is sensitive to parameter selection, such as cost, gamma, and epsilon. It may also struggle with high-dimensional or time-related data if not tuned well. The leftover autocorrelation in the SVRX model indicates it did not capture the temporal patterns, particularly when the data is irregular and noisy (Liu et al., 2021; Haider et al., 2019).

The ANNX model helps farmers reduce fall armyworm problems by using preventive strategies. This includes changing irrigation schedules, timing insecticide applications well, and choosing maize varieties that resist pests. These methods reduce the number and severity of fall armyworm attacks. Agricultural consultancy services simplify the model’s detailed forecasts into clear recommendations for farmers. These services offer regular updates based on model predictions, giving farmers timely advice on when to apply preventive measures for the best results.

These findings highlight the importance of machine learning techniques, especially ANN models with outside inputs, for predicting pests in complex agroecological systems. Adding climate-sensitive models like ANNX into decision support tools can significantly improve pest management strategies. This helps farmers take preventive and timely action against pest outbreaks in changing climate conditions.

## Conclusions

This study used count time series data and machine learning techniques to develop prediction models for fall armyworm occurrences that include climate-related variables. The results show that the data’s diverse and non-linear structure makes both the INGARCHX and SVRX models unsuitable for predicting fall armyworm time series. In contrast, the results demonstrate that the ANNX model is a reliable and effective method for simulating and predicting the occurrence of fall armyworms in time series data. Additionally, the research suggests that using machine learning approaches, like ANN with extra variables, improves the accuracy of count-based time series predictions. The Diebold-Mariano test statistics further confirm the ANNX model’s better performance than the INGARCHX and SVRX models.

## Data Availability

The raw data supporting the conclusions of this article will be made available by the authors, without undue reservation.
